# Essential Oils for Flea and Tick Control in Companion Animals: A Critical Review of Efficacy, Safety, Resistance Mitigation and Integrated Pest Management

**DOI:** 10.3390/antibiotics15030312

**Published:** 2026-03-19

**Authors:** Roberto Bava, Rosa Maria Bulotta, Fabio Castagna, Stefano Ruga, Carmine Lupia, Filomena Conforti, Giancarlo Statti, Rosalia Crupi, Vincenzo Musella, Ernesto Palma

**Affiliations:** 1 Department of Health Sciences, University of Catanzaro Magna Græcia, 88100 Catanzaro, Italy; 2 Mediterranean Ethnobotanical Conservatory, 88054 Sersale, Italy; 3 Department of Pharmacy, Health and Nutritional Sciences, University of Calabria, 87036 Cosenza, Italy; 4 Department of Veterinary Sciences, University of Messina, 98168 Messina, Italy

**Keywords:** essential oils, acaricide resistance, insecticide resistance, biopesticides, ticks, fleas, companion animals, integrated pest management, one health

## Abstract

**Background:** The control of fleas and ticks in companion animals is a persistent challenge with animal welfare and public health implications. The increasing resistance to antiparasitic treatments, coupled with concerns over the environmental impact and non-target effects of synthetic acaricides, has driven interest in sustainable alternatives. Essential oils (EOs) have emerged as potential candidates due to their complex chemistry and modes of action. **Methods:** This review critically analyzes the scientific literature on essential oils for ectoparasite control in companion animals. Specifically, it examines their chemical composition, multi-target mechanisms of action, laboratory and field efficacy, role in resistance mitigation, and integration into IPM strategies. **Results:** Several EOs, particularly those rich in phenolic compounds (thymol, carvacrol, eugenol, and cinnamaldehyde), demonstrate promising in vitro insecticidal and acaricidal activity. Their multi-target mechanisms, affecting neuronal, respiratory, and cuticular functions, not only provide efficacy but also represent a significant barrier to rapid resistance development. However, their translation to reliable field performance is hampered by high volatility, formulation instability, and innate variability. **Conclusions:** EOs represent a valuable source of bioactive compounds for reducing reliance on conventional acaricides and can play a key role within IPM strategies. To realize their full potential in mitigating resistance, focused advancements are needed in standardized testing, formulation science to enhance stability and residual activity, and rigorous field studies to confirm safety and efficacy.

## 1. Introduction

One of veterinary medicine’s ongoing challenges is controlling ectoparasites in companion animals, which has considerable implications for both public health and animal care. Fleas and ticks are not just nuisance pests but also serve as vectors for numerous zoonotic infections, making their efficient control a crucial component of preventive veterinary care [1,2]. Synthetic chemical insecticides and acaricides, such as organophosphates, pyrethroids, neonicotinoids, phenylpyrazoles, and, more recently, isoxazolines, represent the backbone of traditional control methods [3,4]; however, their exceptional efficacy has been accompanied by serious concerns, as their extensive and sometimes indiscriminate usage has led to the growth of resistant parasite populations, environmental pollution concerns, and potential adverse effects on non-target organisms [5,6].

In light of this, there has been increasing interest in investigating alternative control methods that support sustainable pest management and One Health. Aromatic plant-derived essential oils (EOs) have become attractive options due to their potential benefits, which include reduced environmental persistence, lower mammalian toxicity, and numerous modes of action that could prevent the development of resistance [7,8]. These natural products are complex, volatile mixtures of secondary plant metabolites with documented roles in plant defense [9,10].

The scientific exploration of EOs for ectoparasite management has progressed considerably over the past two decades, although the knowledge regarding their practical application remains fragmented. This paper critically assesses the current state of knowledge about essential oils (EOs) and their bioactive components for managing fleas and ticks in companion animals. It addresses the potential and drawbacks of these botanical products and highlights important research gaps that need to be filled before widespread clinical adoption can be advised. The rationale for conducting this review at this time is supported by a convergence of evidence and concern: over the past two decades, several peer-reviewed studies have documented in vitro acaricidal and insecticidal activity of EO preparations against the principal ectoparasites of companion animals, and a subset of these studies has reported susceptibility data confirming retained activity against acaricide-resistant strains of *Rhipicephalus sanguineus* and *Ctenocephalides felis*. At the same time, documented risks have accumulated, including well-characterized neurotoxic events in cats following exposure to phenol-rich EOs (particularly those containing thymol, carvacrol, and terpinen-4-ol), inconsistent field efficacy attributable to inherent formulation volatility and compositional variability, and regulatory gaps that have permitted incompletely characterized products to reach the companion animal market without rigorous efficacy or safety evaluation. This body of evidence (both promising and cautionary) justifies a critical synthesis that clearly distinguishes genuine scientific potential from overstated commercial claims, and that provides actionable guidance for both future research priorities and responsible clinical communication to veterinarians and pet owners.

## 2. Methods

A multistage literature search strategy was adopted, combining structured queries in primary databases with subsequent manual exploration, to ensure adequate breadth and depth of coverage.

In the first phase, literature searches were conducted in PubMed/MEDLINE and Scopus, covering the period from January 2000 to December 2024, with a final update performed in January 2026 to capture the most recent publications, including early online releases. The search string, applied to title, abstract, and keywords fields, utilized the following Boolean combination: (“essential oil” OR “essential oils” OR “botanical acaricide”) AND (flea OR tick) AND (dog OR cat OR pet). This query yielded approximately 40 records from PubMed and 74 records from Scopus. The combined structured search on PubMed and Scopus identified a total of 114 records. From these, 50 original studies were selected as being directly relevant to the scope of this review.

In a second phase, to broaden the literature coverage and include contributions not indexed or not captured by the formal search string, an additional exploratory search was performed on Google Scholar. In this case, a complex Boolean string was not applied; instead, more flexible combinations of keywords (such as “essential oils dog’s fleas”, “plant-based acaricides cats ticks”) were employed, and the first pages of results were examined non-systematically, together with the reference lists of key articles and previously identified reviews. These additional references, identified through the examination of key article bibliographies and targeted searches on Google Scholar, were added to the corpus of the literature, consistent with the exploratory and non-systematic approach of a narrative review.

Original research studies (in vitro and in vivo) published in peer-reviewed journals in the English language were considered eligible for inclusion in the final synthesis, provided they reported data on the efficacy, safety, or mechanisms of action of essential oils or their isolated compounds against fleas and ticks infesting dogs and cats. Non-peer-reviewed sources (theses, conference proceedings, preprints), book chapters, and case reports were excluded. Review articles were consulted exclusively for background information and for the identification of further primary sources, but they are not included in the analysis of experimental data. These 50 studies from the structured search were then supplemented with additional references identified through manual exploration of Google Scholar and key article bibliographies. The combination of structured database searches and manual exploration led to the selection of the 153 references that constitute the final bibliography of this work, deemed representative of the available literature and suitable for an in-depth and critically oriented narrative review.

## 3. Biological Basis for Essential Oil-Based Ectoparasite Control

Evaluating the possible role of EOs in control programs requires an understanding of the biological traits of the target ectoparasites. Fleas, particularly *Ctenocephalides felis*, represent the most prevalent ectoparasite affecting companion animals globally [11]. The life cycle of the cat flea is holometabolous, consisting of egg, larva, pupa, and adult stages. Only adult fleas are obligate hematophagous ectoparasites [12]. Adults only comprise a small portion of the flea population in an infested environment; juvenile stages (eggs, larvae, and pupae) survive in carpets, beds, and other protected microhabitats. This aspect of flea biology is crucial for management strategies [13,14], as the environmental reservoir presents a persistent risk of reinfestation.

The medical and veterinary significance of *C. felis* extends beyond the direct effects of blood feeding. This species is the main culprit for flea allergy dermatitis in dogs and cats, a hypersensitivity reaction that is among the most prevalent skin conditions in pets [15]. Moreover, *C. felis* serves as a vector for *Rickettsia felis*, the causative agent of flea-borne spotted fever in humans, and *Bartonella henselae*, responsible for cat scratch disease. The flea also functions as an intermediate host for *Dipylidium caninum*, a cestode that can occasionally infect children through accidental ingestion of infected fleas [16].

Ticks pose a different but no less serious problem. Species such as *Rhipicephalus sanguineus*, *Ixodes scapularis*, *Dermacentor variabilis*, and various *Amblyomma* species parasitize companion animals across different geographical regions [17,18]. Unlike fleas, the majority of hard ticks are multi-host ectoparasites; to complete their life cycle, many common species require two or three separate hosts. The majority of their life cycle takes place off the host, and they spend extended periods of time in the environment between blood feeding. This life cycle influences both infestation patterns and the control measures required.

Ticks are vectors for a diverse array of pathogens, including *Ehrlichia* species, *Anaplasma* species, *Borrelia burgdorferi*, *Rickettsia* species, and *Babesia* species, making their control critical for preventing vector-borne diseases in both animals and humans [19,20].

When assessing EO efficacy against ticks, it is crucial to consider the varying vulnerability of different developmental stages. Larval ticks are consistently more sensitive to EOs than nymphs and adults, which is probably due to variations in body size in relation to surface area, cuticular thickness, and lipid content [21,22]. This finding has significant implications for control strategies, indicating that treatments meant to kill adult ticks on the host may not be as effective as environmental applications that target questing larvae. Otherwise, adult fleas demonstrate varying susceptibility depending on the oil tested, eggs and pupae are generally more resistant than larvae and newly emerged adults [23]. EO-based treatments would need to either show sustained residual activity to kill emerging adults or be paired with growth regulators to prevent completion of the life cycle, given the relative resistance of eggs and pupae [24]. The concept of life cycle inhibition provides a more thorough evaluation of EO efficacy than mortality assays focusing solely on adults, which includes impacts on oviposition, egg viability, larval development, pupation success, and adult emergence [23,25]. This comprehensive approach has been assessed in a number of investigations, which have shown that several EOs can interfere with the flea life cycle at multiple stages. For example, *Piper aduncum* oil rich in dillapiole showed the capacity to inhibit larval development, lower egg hatchability, and reduce egg production in surviving female fleas, resulting in an overall life cycle inhibition exceeding 90%, even though it only caused moderate adult mortality [26].

The need to create sustainable management measures is increased by the growing awareness of how climate change affects ectoparasite distributions. Both fleas and ticks are expanding their geographic ranges, extending their seasonal activity periods, and possibly raising the risk of disease transmission due to environmental changes. This shifting ecosystem necessitates adaptable, environmentally adaptive approaches to ectoparasite management that can be tailored to changing conditions without contributing to environmental degradation or resistance development.

## 4. Chemical Composition, Variability, Mechanisms of Action, and Acaricidal/Insecticidal Activity of Essential Oils

Essential oils (EOs) are concentrated, volatile, lipophilic mixtures of secondary plant metabolites extracted from aromatic tissues [27]. Their chemical composition is highly variable, depending on plant species, genotype, chemotype, plant organ, geographical origin, harvest time, extraction method, and storage conditions [28,29]. This intrinsic heterogeneity represents a major challenge for standardization but also an opportunity to identify highly active chemotypes, as bioactivity is profoundly influenced by chemical composition. A clear example is provided by *Ageratum conyzoides*: the white-flower chemotype, dominated by precocene at 80.4%, showed minimal activity against *Rhipicephalus microplus* larvae, whereas the purple-flower chemotype, rich in sesquiterpenes such as β-acoradiene and γ-amorphene, exhibited substantial acaricidal potency with an LC_50_ of 1.49 mg/mL [30]. Such differences underscore the critical need for thorough phytochemical characterization and deliberate chemotype selection when developing reliable botanical acaricides. The principal constituents of EOs derive from two major biosynthetic pathways: terpenoids, including monoterpenoids and sesquiterpenoids, and phenylpropanoids [31]. Among monoterpenoids commonly associated with insecticidal and acaricidal activity are carvacrol, thymol, menthol, 1,8-cineole, linalool, α-pinene, and limonene [32]; these are typically low-molecular-weight compounds below 300 Da and highly volatile. Sesquiterpenoids, such as nootkatone and β-caryophyllene, are less volatile and often contribute to residual activity [33]. Phenylpropanoids such as eugenol, cinnamaldehyde, and methyl eugenol dominate EOs from Lauraceae and Myrtaceae families and frequently display strong insecticidal effects [34]. The biological activity of EOs against arthropods stems from complex, multi-target mechanisms, a form of polypharmacology that contrasts with the single-target action of many synthetic insecticides. This broad-spectrum activity likely contributes to a slower development of resistance. Empirical support for the role of EOs in managing resistant populations comes from studies comparing susceptible and field-resistant strains. For instance, essential oils from *Schinus molle* and *Bulnesia sarmientoi*, as well as their components (guaiol, bulnesol), maintained significant efficacy against a *R. microplus* field population with documented resistance to pyrethroids and fipronil, although higher concentrations were required compared to a susceptible laboratory strain. This demonstrates that while cross-resistance or tolerance may exist, the multi-target action of EOs can remain effective [35]. Documented modes of action include interference with neurochemical transmission through modification of acetylcholinesterase activity, interaction with GABA-gated and glutamate-gated chloride channels, modulation of octopamine and tyramine receptors, and inhibition of glutamate-gated chloride channels [36,37], as well as alteration of cuticular permeability leading to desiccation, disruption of detoxification enzyme systems, impairment of growth, development, and reproduction, respiratory toxicity via spiracle blockage or fumigant effects particularly with volatile monoterpenes [38,39], and olfactory-mediated repellent effects. Respiratory toxicity was demonstrated in *Ixodes ricinus* nymphs exposed to tea tree oil (*Melaleuca alternifolia*) vapors, with mortality correlating to exposure time (≥90 min) rather than dose, suggesting spiracle blockage or neurotoxicity via the respiratory route [40].

Specific molecular insights are emerging: carvacrol and isoeugenol act as positive allosteric modulators of the tyramine receptor (RmTAR1) in *Rhipicephalus microplus*, significantly enhancing the cellular response to endogenous tyramine [41]. This supports G-protein-coupled receptors such as tyramine receptors as promising targets for EO-inspired rational acaricide design. A particularly intriguing pharmacological feature is the frequent observation of synergistic interactions among EO constituents: whole oils are often more potent than their dominant compounds alone, suggesting that minor components facilitate penetration, inhibit detoxification, or produce additive effects at multiple sites [42,43]. Quantitative analysis of binary mixtures illustrates both the potential and the complexity of this phenomenon. Combinations of lemongrass and white thyme oils showed considerable synergistic effects against adult *Ixodes scapularis* at various doses, yielding an LC_50_ (18.5 μg/μL) comparable to that of highly active white thyme oil alone, whereas combinations of geranium and savory thyme or lemongrass and geranium often showed antagonistic effects, decreasing overall effectiveness compared to the individual oils [44]. These findings underscore that synergistic potential cannot be presumed and must be empirically evaluated for each target species and combination, enabling the rational design of multi-botanical formulations.

## 5. Evidence for Acaricidal and Insecticidal Activity

The study of essential oils (EOs) against ticks and fleas in companion animals has revealed substantial diversity in activity depending on parasite species, developmental stage, botanical family, chemotype, and formulation. Among ticks, the Lamiaceae family (*Origanum, Thymus, Rosmarinus, Salvia*) has received the most attention, with many studies demonstrating notable acaricidal effects [45]. *Origanum vulgare* EO, rich in carvacrol and thymol, has shown remarkable activity against *Rhipicephalus microplus*, although efficacy varies with chemotype and tick population [46,47].

Systematic evaluations indicate that Lauraceae species, particularly *Cinnamomum* spp. rich in cinnamaldehyde and eugenol, exhibit broad-spectrum tick-killing activity across multiple species, including *R. sanguineus*, *R. microplus*, *Dermacentor* spp., and *Amblyomma* spp. [48,49,50]. The proposed mechanisms for these oils align with the multi-target actions described in Section 4, including cuticular disruption and neurotoxicity.

Myrtaceae oils, especially from *Melaleuca alternifolia* (tea tree) and *Eucalyptus* spp., show laboratory activity, though generally less potent than Lamiaceae or Lauraceae oils; their main monoterpenoids (terpinen-4-ol, 1,8-cineole) act primarily via respiratory interference and cuticular disruption [51,52]. Rutaceae oils from citrus species, rich in D-limonene, show activity, but their high volatility often limits residual efficacy [53].

The quantification of acaricidal efficacy further highlights the role of specific chemotypes. For instance, *Tetradenia riparia* (Lamiaceae) EO demonstrated strong activity against *R. microplus*, with LC_50_ values of 0.534 mg/mL for engorged female mortality and 1.222 mg/mL for larvae, achieving 100% engorged female death at 1.8% concentration [54]. Analysis of *Lippia gracilis* genotypes showed a strong chemotype–activity correlation, with carvacrol-rich oils superior; isolated carvacrol was significantly more potent (LC_50_ = 0.22 mg/mL against larvae) than thymol (LC_50_ = 3.86 mg/mL) [55].

Beyond Lamiaceae, *Cuminum cyminum* and *Pimenta dioica* EOs achieved 100% mortality of *R. microplus* larvae at low concentrations (2.5% and 1.25%), likely due to cuminaldehyde and methyl eugenol [56]. Consistent with this trend, isolated eugenol, the dominant compound in clove and certain basil oils, showed significant efficacy against *R. sanguineus* larvae (LC_90_ = 14.8 mg·mL^−1^), outperforming the whole *O. gratissimum* oil (LC_90_ = 46.8 mg·mL^−1^) [57]. This finding suggests that the activity of the complex natural mixture can sometimes be exceeded by isolated key phenolic compounds. Importantly, the activity of certain EOs extends to acaricide-resistant tick strains. Luns et al. (2021) [35] showed that *Bulnesia sarmientoi* oil and its constituents guaiol and bulnesol were effective against a multi-resistant *R. microplus* field population, highlighting their potential as part of resistance management strategies.

Research on fleas, predominantly *Ctenocephalides felis,* due to their global prevalence and laboratory maintainability [58], shows that EOs from certain families consistently exhibit strong insecticidal effects across multiple developmental stages. Lamiaceae oils rich in phenolic monoterpenoids are particularly effective against adult and immature stages [32].

In contact bioassays, EOs from *Thymus vulgaris* and *Origanum vulgare*, rich in thymol and carvacrol, yield lethal concentrations in the low microgram per milliliter range against adult *C. felis* [59]. Beyond adulticidal action, these compounds and whole oils also display significant ovicidal and larvicidal activity, showing potency against vulnerable first-instar larvae [60]

Phenylpropanoid-rich EOs from Lauraceae and Myrtaceae often show superior efficacy: *Cinnamomum* spp. rich in cinnamaldehyde are lethal to adult fleas at lower doses than thymol or carvacrol [61] while eugenol-rich *Syzygium aromaticum* oil exerts potent adulticidal and developmental inhibitory effects [59]. Mechanisms for these phenylpropanoids include interference with octopaminergic signaling and acetylcholinesterase inhibition, with additional pathways contributing to toxicity [62]. A direct comparative study of cinnamon (*Cinnamomum cassia*), thyme (*Thymus vulgaris*), and oregano (*Origanum vulgare*) EOs against all life stages of *C. felis* provided valuable quantitative insights: cinnamon oil (91% cinnamaldehyde) was most effective against immature stages, with the lowest LC_50_ values against eggs (3.0 μg·cm^−2^), larvae (10.3 μg·cm^−2^ at 48 h), and pupae (34.6 μg·cm^−2^), and the highest overall development inhibition (LC_50_ = 2.3 μg·cm^−2^); oregano oil (76.2% carvacrol) showed the greatest adulticidal activity (LC_50_ = 21.8 μg·cm^−2^ at 48 h); thyme (44.7% thymol) and oregano oils displayed the most promising residual activity in vitro, retaining >80% mortality for up to 6 days post-application [63]. This chemotype-dependent and stage-specific profile is essential for guiding targeted control strategies.

Comparative patterns across ticks and fleas highlight consistent themes: phenolic monoterpenoids (carvacrol, thymol) and phenylpropanoids (cinnamaldehyde, eugenol) dominate efficacy; larval stages are generally more susceptible than nymphs/adults in ticks and eggs/pupae in fleas, likely due to differences in surface area-to-volume ratio, cuticular thickness, and lipid content; life cycle inhibition (oviposition reduction, egg hatchability decrease, larval development block) often exceeds adult mortality alone, as seen with *Piper aduncum* oil rich in dillapiole (>90% overall inhibition despite moderate adult kill) [26]. These findings emphasize the need for comprehensive bioassays evaluating impacts across all life stages rather than adult mortality alone.

However, a recent field study demonstrated that an oral spray containing a mixture of garlic, rapeseed, and rosehip oils (Lacecca^®^) achieved 100% efficacy against *Rhipicephalus sanguineus* on naturally infested dogs within 12 h of the third daily dose, with protection lasting 21 days and no adverse effects on hematological or biochemical parameters [64]. This provides rare in vivo evidence for the potential of orally administered EO blends, although the specific contribution of each oil and the mechanism of systemic action require further investigation.

### 5.1. In Vitro Insecticidal Activity Against Fleas

The pulicidal activity of several essential oils has been widely demonstrated in vitro. In a comparative study evaluating the effect of six oils on multiple stages of *Ctenocephalides felis felis*, Dos Santos et al. [23] identified *Ocimum gratissimum* oil as the most promising. This oil, rich in eugenol (74.5%), showed significantly lower CL50 values for adults, eggs and larvae (5.85, 1.79 and 1.21 μg/cm^2^, respectively) than other oils tested, such as those of *Cinnamomum* spp. or *Laurus nobilis*. The study also found that larvae are the most susceptible stage, which is important for targeting treatments. In parallel, Batista et al. [65] demonstrated that the choice of the plant part and the extraction solvent is crucial for pulicidal activity. Testing *Schinus molle*, the authors found that leaf oil (with main components such as spathulenol and cubenol) was much more active (LC50 12.02 μg/cm^2^) than fruit oil (LC50 353.95 μg/cm^2^) against adult fleas. In addition, the hexane extract (non-polar) was the only active extract, suggesting that the active ingredients responsible for the insecticidal effect are non-polar in nature. Interestingly, despite its potent activity against fleas, *S. molle* oil showed low toxicity on a eukaryotic model (*Saccharomyces cerevisiae*), suggesting a potential higher safety profile for mammalian hosts.

Finally, a study of *Baccharis trimera* and *Mimosa verrucosa* showed activity against all flea life stages [66]. *B. trimera* (rich in carquejile acetate) showed marked larvicide activity (CL90 44.9 μg/cm^2^), while *M. verrucosa* (rich in β-pinene) was more effective on eggs (CL90 not available, but overall efficacy was higher). Both oils showed a residual effect limited to only three days [66]. The full data from these studies are summarized in Table 1.

### 5.2. In Vitro Acaricide Activity Against Ticks of the Genus Rhipicephalus

Numerous in vitro studies have documented the acaricide activity of various essential oils against ticks of the genus *Rhipicephalus* (Table 2). Tadee et al. [67] tested several oils, finding that clove oil was the most effective against larvae (LC90 32.06 ppm at 24 h), with larval mortality of more than 99% at concentrations ≥2% and the ability to inhibit oviposition at higher concentrations (16%), with minimal adverse effects on canine skin.

Studies on *Thymus vulgaris* oil have shown that the nanoemulsion formulation significantly increases its effectiveness. Alibeigi et al. [68] reported a CL50 for larvae of 0.09% for nanoemulsion, a much lower value than for pure oil. The same study also confirmed that thymol, the main component, is effective in reducing oviposition in females (44.9% at 20 mg/mL). Another study [69] combined thymus nanoemulsions with silver nanoparticles (TNE-AgNPs), obtaining the highest toxicity (CL50 2.38% at 7 days) and showing significant morphological damage to ticks, such as alterations of sensilli and aeropiles, under the electron microscope.

Duarte et al. [70] tested patchouli, palmarosa, and lemongrass oils on *Rhipicephalus linnaei*, finding that nymphs are more sensitive than larvae. Patchouli oil was the most effective, with a CL90 of 2.21 mg/mL on larvae and 100% mortality on nymphs at 5 mg/mL. The study also highlighted how efficacy can vary between private labels, underlining the importance of a standardized chemical composition.

Pereira et al. [71] showed that *Egletes viscosa* (rich in cis-isopinocarveyl acetate) and *Lippia schaueriana* (rich in piperitenone oxide) oils, while not very lethal for adult females (control efficacy of 92.7% and 84.6% respectively at 50 mg/mL), significantly reduced their reproductive capacity, with an inhibition of oviposition of up to 62.7% for *E. viscosa*. The eggs laid appeared dehydrated and dark, suggesting an effect on Gene’s organ.

Other studies have confirmed the larvicide activity and sub-lethal effects of oils such as *Schinus molle* [72] and *Melaleuca cajuputi* [73], while *Achyrocline satureioides* oil has shown more modest activity in the first study on the species against *R. sanguineus* [74].

**Table 2:** In vitro efficacy of essential oils and components against ticks of the genus *Rhipicephalus*.

Target Species (Parasite)	Assay Method	Essential Oil/Component	Main Component (If Specified)	LC50/LC90 or % Efficacy	Tested Concentrations	Reference
*Rhipicephalus sanguineus*, *Ctenocephalides felis*	Adult Immersion Test (AIT), Larval Immersion Test, Flea mortality test	*Zanthoxylum limonella*, Citronella, Clove, Peppermint, Ginger	Not specified	LC90 (larvae, 24 h): 32.06–98.26 ppm; LC90 (fleas, 1 h): 19,274–425,045 ppm; Larval mortality >99% at ≥2%.	0.5%, 1%, 2%, 4%, 8%, 16%	[67]
*Rhipicephalus sanguineus*	Larval mortality test in microtube, Adult Immersion Test (AIT)	*Thymus vulgaris*—Oil, Nanoemulsion, Thymol, p-Cymene	Thymol (38.4%), γ-Terpinene (15.1%), p-Cymene (18.5%)	LC50 (larvae, nano EO): 0.09%. Oviposition inhibition (AIT): Thymol 44.9% at 20/40 mg/mL.	0.125–4% (EO/nano); 2.5–40 mg/mL (Thymol, p-Cymene)	[68]
*Rhipicephalus sanguineus*	Adult Immersion Test (AIT), Scanning Electron Microscopy (SEM)	*Thymus vulgaris*—Oil, Nanoemulsion (TNE), Silver Nanoparticles (AgNPs), TNE+AgNPs	Thymol (34.1%), γ-Terpinene (33.0%)	LC50 (7 days): Thyme oil 11.62%; TNE 5.47%; AgNPs 4.08%; TNE-AgNPs 2.38%.	Thyme oil: 5–40%; TNE: 2.5–30%; AgNPs/TNE-AgNPs: 1–5%	[69]
*Rhipicephalus linnaei* (= *R. sanguineus* s.l.)	Immersion Test (IT) on larvae and nymphs	*Pogostemon cablin*, *Cymbopogon martinii*, *Cymbopogon flexuosus*	Patchouli: patchouli alcohol; Palmarosa: geraniol; Lemongrass: neral, α-citral	LC90 (larvae, patchouli): 2.21 mg/mL. Larval mortality (patchouli): 100% at 10 mg/mL. Nymphal mortality (patchouli): 100% at 5 mg/mL.	2.5, 5, 10, 20 mg/mL	[70]
*Rhipicephalus sanguineus* s.l.	Adult Immersion Test (AIT)	*Egletes viscosa*, *Lippia schaueriana*	*E. viscosa*: cis-isopinocarveyl acetate (68.4%); *L. schaueriana*: piperitenone oxide (64.4%), Limonene (21.3%)	Control efficacy (AIT, 50 mg/mL): *E. viscosa* 92.7%; *L. schaueriana* 84.6%. Oviposition inhibition: up to 62.7% (*E. viscosa*).	12.5, 25, 50 mg/mL	[71]
*Rhipicephalus sanguineus* (larvae)	Larval Packet Test (LPT)	*Achyrocline satureioides*—Crude ethanolic extract and Essential Oil	Oil: terpenes (α-pinene, β-myrcene, D-limonene, etc.)	LC50/LC90 (oil): 119.7/185.5 mg/mL. Mortality at 100 mg/mL: Oil 56.6%.	0.78–100 mg/mL	[74]
*Rhipicephalus sanguineus*	Larval Packet Test (LPT), Adult Immersion Test (AIT)	*Schinus molle*	p-cymene (40.0%), limonene (19.5%), myrcene (7.7%)	LC50/LC90 (larvae): 0.21/0.80%. Effect on reproduction (20%): Oviposition inhibition 29.6%; Reproductive efficiency 22.6.	Larvae: 0.125–2%; Adults: 0.125–20%	[72]
*Rhipicephalus sanguineus*	Larval Packet Test (LPT), Adult Immersion Test (AIT)	*Melaleuca cajuputi*	α-Terpineol (41.9%), Eucalyptol (5.8%), β-Linalool (5.6%)	LD50/90/99 (larvae): 1.27 / 8.96 / 43.95 mg/mL. Production inhibition (EP%): 42.8% at LD50.	0.3125–20 mg/mL (larvae); LD50 (for adults)	[73]
*Rhipicephalus sanguineus* s.l. (larvae)	Larval Immersion Test (LIT)	*Cedrus libani* (Tar)	β-himachalene (29.2%), α-atlantone (28.7%), ar-turmerone (8.8%)	LC50/LC90 (larvae): Kepez strain 0.47/1.52%; Konyaalti strain 0.58/1.63%.	0.1%, 0.5%, 1%	[75]
*Rhipicephalus sanguineus* (larvae and nymphs)	Larval/Nymphal Packet Test	*Lippia sidoides*	Thymol (69.9%), o-cymene, E-caryophyllene	LC90 (R. sanguineus): Larvae 11.56 mg/mL; Nymphs 12.97 mg/mL.	2.35, 4.70, 9.40, 14.10, 18.80 mg/mL	[76]

**Note:** Concentration units are presented unchanged from the source publications to preserve fidelity. Differences reflect assay methodology: immersion/larval packet tests commonly report % (*w*/*v*), ppm, or mg/mL; filter-paper/contact assays report μg/cm^2^. Readers should consult individual references for exact definitions.

### 5.3. Acaricide Activity of Pure Components and Synergistic Mixtures

The analysis of pure components and their mixtures has made it possible to identify the main culprits of acaricide activity and to exploit synergistic effects (Table 3). Araújo et al. [77] determined for the first time the CL50 values of thymol, carvacrol and eugenol against *R. sanguineus*, with thymol being the most active (2.98 mg/mL). The study showed that most binary combinations produce synergistic effects (combination index, CI < 0.70), in particular those containing eugenol.

Oil blends, such as mint and clove, also showed a significant synergistic factor (synergistic factor 2.46) against larvae [78]. In contrast, components such as linalool have been shown to be poorly effective on their own, causing only 7.3% mortality at 2.5 μL/mL [79]. *Origanum minutiflorum* oil (rich in carvacrol) has been shown to be an extremely effective larvicide on its own, with a CL50 of 0.10% [80].

**Table 3:** In vitro efficacy of pure components and synergistic mixtures against ticks of the genus *Rhipicephalus*.

Target Species (Parasite)	Assay Method	Essential Oil/Component	Main Component (If Specified)	LC50/LC90 or % Efficacy	Tested Concentrations	Reference
*Rhipicephalus sanguineus* s.l. (larvae)	Larval Repellent Activity Test (LRAT)	*Origanum minutiflorum*, *Dorystoechas hastata*	*O. minutiflorum*: carvacrol (>80%); *D. hastata*: borneol, camphor, 1,8-cineole	LC50/LC90 (larvae): *O. minutiflorum* 0.10/0.13%; *D. hastata* 0.94/2.1%.	0.075–3%	[80]
Dog ticks (*R. sanguineus*, *A. cajennense*)	Larval/Nymphal Packet Test	Carvacrol, (E)-cinnamaldehyde, trans-anethole, linalool	Carvacrol, Cinnamaldehyde, Anethole, Linalool	Mortality at 2.5 μL/mL on *R. sanguineus*: Carvacrol 100%; Cinnamaldehyde 100%; Anethole 7.3%; Linalool 0%.	2.5, 5.0, 10.0, 15.0, 20.0 μL/mL	[79]
*Rhipicephalus sanguineus*	Larval Immersion Test (LIT), Adult Immersion Test (AIT)	*Syzygium aromaticum*, *Mentha longifolia*, *Pelargonium graveolens*—oils, binary combinations, and nanoemulsions	Clove: eugenol (68.0%); Mint: pulegone (33.5%); Geranium: citronellol (22.2%)	LC50 larvae: Mint NE 0.36%; (Clove+mint) 1.43%. LC50 adults: Clove NE 1.63%; (Mint+Geranium) 4.93%. Synergistic Factor (SF): (Clove+mint) 2.46 on larvae.	Oils/combinations: 0.625–20%; NE: 0.23–15%	[78]
*Rhipicephalus sanguineus* (larvae)	Larval Packet Test (LPT), synergism analysis	Thymol, Carvacrol, Eugenol (binary combinations)	Thymol, Carvacrol, Eugenol	LC50 (R. sanguineus): Thymol 2.98 mg/mL; Carvacrol 3.29 mg/mL; Eugenol 5.19 mg/mL. CI: 8/9 combinations <0.70 (synergism).	0.31–7.5 mg/mL; and fractions of LC50	[77]

**Note:** Concentration units are presented unchanged from the source publications to preserve fidelity. Differences reflect assay methodology: immersion/larval packet tests commonly report % (*w*/*v*), ppm, or mg/mL; filter-paper/contact assays report μg/cm^2^. Readers should consult individual references for exact definitions.

### 5.4. Development and Evaluation of Formulations

The development of advanced formulations is crucial to overcome the limitations of essential oils, such as their volatility and poor stability. As shown in Table 4, nanoemulsions represent a promising strategy. Abdel-Ghany et al. [81] have shown that nanoemulsions of myrrh, cypress and patchouli have greater efficacy (lower CL50: 4.17%, 5.04% and 8.57% at 7 days, respectively) than pure oils, although with a slower effect. The same study also confirmed low toxicity of these formulations on human fibroblast cells at effective concentrations. 

Another formulation development study [66] tested spray and spot-on formulations based on eugenol and 10% carvacrol. Formulations with the combination of the two active ingredients showed a faster knockdown effect (100% in 6 h for spot-on), indicating a synergistic effect. However, residual efficacy against ticks was poor (only 2–3 days), highlighting the need for further improvements to prolong activity over time.

### 5.5. In Vivo Studies on Dogs

Although most studies are in vitro, some research has validated the effectiveness of essential oil products directly on dogs (Table 5). An innovative study by Amer & Amer [64] showed that an oral mixture of garlic, rapeseed and rosehip oils achieved^®^ 100% efficacy in the prevention and 99.4% in the treatment of *Rhipicephalus sanguineus* infestations, with an improvement in hematological parameters and no adverse effects.

A clinical trial with a poly-herbal oil made from lemongrass, lemongrass, and mint in sesame oil showed good efficacy (75% recovery for ticks/fleas/lice and 87.5% for mange), although lower than the chemical control amitraz. Improvements in hematological and biochemical parameters were also observed [82]. Another integrated approach (treatment of the animal and the environment) with herbal mixtures has shown excellent and long-lasting efficacy [83].

Finally, Várguez-Tec et al. [84] showed that a simple concentrated aqueous extract of green anise seeds (*Pimpinella anisum*) caused 100% of ticks to detach in vivo in a significantly shorter time (60.8 min) than amitraz (145.1 min), identifying p-anisaldehyde as the probable active ingredient responsible for immobilization.

**Table 5:** In vivo efficacy of essential oils and their extracts on parasite-infested dogs.

Target Species (Parasite)	Assay Method	Essential Oil/Component	Main Component (If Specified)	LC50/LC90 or % Efficacy	Tested Concentrations	Reference
Ticks (mainly *R. sanguineus*)	Artificial infestation and in vivo counting	Oral essential oil blend (Lacecca^®^: garlic, allicin, rapeseed oil)	Allicin, sulfur compounds from garlic	Efficacy (%): 100% in prevention of infestation; 99.43% in treatment of infestation at 28 days.	0.25 mL/kg orally for 3 consecutive days	[64]
*Rhipicephalus sanguineus*, *Ctenocephalides felis*	Topical and environmental application of herbal mixtures	Mixture of Neem, Turmeric, Tulsi, Aloe vera, etc.	Not applicable	Efficacy (%): “Excellent,” with complete parasite removal and no reinfestation for over a year.	Not specified	[83]
Ticks, fleas, lice, mange mites	In vivo clinical study (weekly topical application)	Polyherbal oil (Citronella, Lemongrass, Mint in sesame oil)	Citronellal, Citral, Menthol	Clinical efficacy (ticks/fleas/lice): 75% recovery after 21 days. Clinical efficacy (mange): 87.5% recovery after 35 days.	Mixture of 5:5:5 drops per 10 mL carrier oil	[82]
*Rhipicephalus sanguineus*, *Ixodes affinis*	In vivo test (application on infested dogs)	*Pimpinella anisum*—Aqueous seed extract	p-anisaldehyde (0.55%)	In vivo detachment (100% extract): 100% in 60.8 min (vs. 145.1 min for Amitraz).	In vivo: 100%	[84]

**Note:** Concentration units are presented unchanged from the source publications to preserve fidelity. Differences reflect assay methodology: immersion/larval packet tests commonly report % (*w*/*v*), ppm, or mg/mL; filter-paper/contact assays report μg/cm^2^. Readers should consult individual references for exact definitions.

## 6. Repellent Properties and Their Relevance

### 6.1. Mechanisms and Evaluation Methods

The repellent effect of EOs provides an additional mechanism that could enhance their utility in ectoparasite management, particularly in preventing initial infestations [85]. In arthropod control, repellency is defined as the ability of a substance to cause arthropods to avoid treated surfaces or to detach soon after contact, thereby preventing or reducing attachment and feeding [86]. Even short-term repellency that delays feeding in disease-vector ectoparasites could theoretically reduce pathogen transmission, provided the delay exceeds the minimum attachment time required for pathogen transfer [87].

Laboratory evaluation of EO repellency against ticks employs various techniques, most commonly vertical or horizontal climbing assays, where tick behavior is observed upon encountering treated surfaces [88]. Major components of Cymbopogon oils, citronellal and citronellic acid, have consistently demonstrated potent tick repellent activity, with efficacy comparable to synthetic repellents like DEET in short-term assays [89]. The mechanism appears to involve activation of contact chemoreceptors on the tick’s Haller’s organ, which induces avoidance behavior [90].

Certain EO constituents responsible for repelling effects have been effectively identified using bioactivity-guided isolation investigations. In an investigation of *Pelargonium graveolens* (geranium) essential oils, the sesquiterpene alcohol (-)-10-epi-γ-eudesmol was isolated and found to be a key repellent against *Amblyomma americanum* nymphs, with efficacy similar to DEET at concentrations ≥0.052 mg/cm^2^ [91]. This shows that not only the main compounds, but also minor or mid-level elements might be crucial for bioactivity.

Additionally, screening studies that employ standardized contact-repellency assays offer useful comparison information. A recent test of 20 essential oils packaged in lotion found clove oil, cinnamon oil, and geraniol offer the most sustained total protection period (>1 h) against both *Ixodes scapularis* tick crosses and *Aedes aegypti* mosquito bites [92]. The fact that this strong contact repellency did not necessarily correspond with long-distance olfactory repellency observed in other assay types highlights the fact that different products may have different modes of action and appropriate applications. Repellency is also documented against other genera of veterinary importance. A laboratory study on *Dermacentor reticulatus* adults found that 3% clove bud (*Syzygium aromaticum*) and creeping thyme (*Thymus serpyllum*) essential oils repelled 83% and 82% of ticks, respectively. Furthermore, a mixture of creeping thyme and citronella oils (1.5% each) showed a synergistic effect, achieving 91% repellency [93]. This underscores the potential of optimized EO blends for broad-spectrum protection.

The critical influence of bioassay design on perceived efficacy was recently illustrated in a comparative study of 16 EOs against *Amblyomma americanum* nymphs. Oils like citronella and juniper berries demonstrated excellent repellency in a forced-contact assay but were weak in a spatial (volatile) assay and ineffective in a human fingertip assay. Conversely, 1R-trans-chrysanthemic acid (TCA) and thyme oil were powerful in both contact and spatial experiments, but only TCA transferred well to the fingertip test [94]. This work empirically indicates that repellency is not an intrinsic trait but is defined by the exposure situation. In order to more accurately forecast the efficacy of repellent formulations in the real world, it makes the case for the implementation of tiered testing methodologies, which go from controlled contact to spatial and eventually to host-based assessments. The repellent effect of essential oils represents an important complementary strategy to direct control, and the main results are summarized in Table 6. Goode et al. [89] demonstrated in an in vivo participatory study that 2.5% turmeric oil has comparable repellent efficacy to 20% DEET, with only 15% of treated dogs having ticks in the sprayed areas (paws and belly), compared to 73% of the control group. 

Laboratory studies have identified the most active components. Koc et al. [95] used a novel larval repellency test (LRAT) to demonstrate that carvacrol and geraniol are the most promising repellents, with 2.5% carvacrol achieving an efficacy (75–91.5%) comparable to 15% DEET. The same group also showed that 1% cedar tar (*Cedrus libani*) has up to 100% repellent activity, also comparable to DEET [96]. 

*Backhousia citriodora* oil, rich in citral, has been shown to be an effective repellent against adults of *R. sanguineus* for a period of up to 3 h [85]. A semi-field study of *Rhipicephalus appendiculatus* confirmed that applying 10% *Tagetes minuta* oil to the legs and tail reduced infestation by 76.5%, being more effective than applying it to the ear [97]. Finally, oils of *Baccharis trimera* and *Mimosa verrucosa* have also shown repellent activity against adult fleas, although at high concentrations [98].

**Table 6:** Repellence activity verified in studies.

Target Species (Parasite)	Assay Method	Essential Oil/Component	Main Component (If Specified)	Efficacy/Main Result	Tested Concentrations	Reference
*Ixodes ricinus*	In vitro repellency bioassay, Blanket-drag field assay, Participatory in vivo study	Turmeric oil, Orange oil, DEET	Turmeric: turmerone	In vivo (2.5%): only 15% of treated dogs had ticks in sprayed areas (legs and belly), compared to 73% of controls.	1.25%, 2.5%, 5%	[89]
*Rhipicephalus sanguineus* s.l. (larvae)	Larval Repellent Activity Test (LRAT)	*Origanum minutiflorum*, *Dorystoechas hastata*	*O. minutiflorum*: carvacrol (>80%); *D. hastata*: borneol, camphor, 1,8-cineole	Repellency (3 h, 1%): *O. minutiflorum* 84–100% (comparable to 15% DEET); *D. hastata* max 72%.	0.1%, 0.5%, 1%	[80]
*Rhipicephalus appendiculatus*	Semi-field studies	*Tagetes minuta*, *Tithonia diversifolia* (10% in petroleum jelly)	*T. minuta*: dihydrotagetone, ocimenone	% tick reduction (legs+tail application): *T. minuta* 76.5%; *T. diversifolia* 67.0%.	10% in petroleum jelly	[97]
*Rhipicephalus sanguineus* s.l. (adults)	Choice test (repellent vs. control)	*Backhousia citriodora*, *Callistemon viminalis*, *Cinnamodendron dinisii*	*B. citriodora*: citral (98.9%)	Repellency (RI at 56 μL/mL): *B. citriodora* 0.17. Duration of efficacy: *B. citriodora* active for up to 3 h.	7, 14, 28, 56 μL/mL	[85]
*Rhipicephalus sanguineus* s.l. (larvae)	Larval Repellent Activity Test (LRAT)	Carvacrol, Geraniol, Cineole, α-pinene, γ-terpinene	Carvacrol, Geraniol	Repellency (2.5%): Carvacrol 75–91.5% (comparable to 15% DEET). Repellency (5%): Geraniol 98.6% (1 h).	0.1%, 0.5%, 1%, 2.5%, 5%	[95]
*Rhipicephalus sanguineus* s.l. (larvae)	Larval Repellent Activity Test (LRAT)	*Cedrus libani* (Tar)	β-himachalene (29.2%), α-atlantone (28.7%), ar-turmerone (8.8%)	Repellency (1%): up to 100% (comparable to 15% DEET).	0.1%, 0.5%, 1%	[75]
*Ctenocephalides felis felis* (adult fleas)	Filter paper repellency test	*Baccharis trimera*, *Mimosa verrucosa*	*B. trimera*: carquejyl acetate (33.0%); *M. verrucosa*: β-pinene (14.2%)	Max repellency (40,000 µg/mL): *B. trimera* 80% at 12 h; *M. verrucosa* 75% at 6 h.	5000, 20,000, 40,000 µg/mL	[99]

**Note:** Concentration units are presented unchanged from the source publications to preserve fidelity. Differences reflect assay methodology: immersion/larval packet tests commonly report % (*w*/*v*), ppm, or mg/mL; filter-paper/contact assays report μg/cm^2^. Readers should consult individual references for exact definitions.

### 6.2. Strategy to Enhance Repellence Activity

A significant limitation of most repellency studies is their brief evaluation period, typically ranging from minutes to hours under laboratory conditions. Environmental parameters such as temperature, humidity, airflow, and substrate properties all influence the volatilization rate of EO components, directly affecting the duration of repellent action [100]. Only a limited number of studies have assessed residual repellency over days or weeks, despite such data being crucial for evaluating the practical utility of EO repellents in real-world applications [96].

The development of microencapsulated or polymer-matrix formulations has shown promise for prolonging the repellent efficacy of volatile EO constituents [98]. By inhibiting volatilization while allowing controlled release to maintain bioactivity, controlled-release formulations can sustain effective concentrations of repellent compounds on treated surfaces over extended periods [101]. This strategy has been effectively applied with synthetic repellents and requires a comprehensive study for EO-based repellents targeting ectoparasites. Field trials using treated clothing represent a critical translational step. A study treating cotton trousers with 5% spearmint (*Mentha spicata*) or oregano (*Origanum vulgare*) essential oils demonstrated a significant reduction in *Ixodes ricinus* nymph acquisition during standardized walks in tick-infested habitat, with efficacy comparable to 20% DEET over 24 h [102]. This highlights the practical potential of EO-treated textiles for personal protection.

It is crucial to recognize that while spatial or contact repellency inhibits ectoparasite attachment, it does not reduce the overall parasite population in the environment. Repellent strategies should therefore be considered complementary to, rather than substitutes for, treatments that kill ectoparasites or disrupt their life cycles [103]. Combining insecticidal/acaricidal and repellent properties in a single product offers potential benefits. However, formulation challenges arise in balancing the need for residual persistence (to kill parasites that breach the repellent barrier) with surface volatilization (to generate repellent vapor) [104]. Formulation strategies for prolonging both repellency and lethal activity are addressed in the next section.

## 7. Formulation Challenges and Innovations

### 7.1. Core Formulation Obstacles

Translating promising in vitro activity into viable veterinary products requires overcoming major formulation challenges inherent in the physical and chemical properties of EOs. The high volatility of most EO constituents leads to a rapid loss of active compounds from treated surfaces, significantly reducing residual efficacy [105]. EO stability under field conditions is further compromised by photochemical degradation, particularly of unsaturated terpenoids [106]. The lipophilic nature of EOs complicates the preparation of homogeneous aqueous solutions, and their direct application can cause skin irritation in some species [107].

### 7.2. Conventional and Advanced Emulsion Technologies

Emulsion technology is the most basic method for incorporating EOs into water-based formulations suitable for topical application. Conventional oil-in-water emulsions can be prepared using food-grade surfactants and co-surfactants, although these systems often suffer from instability issues such as creaming, coalescence, and phase separation during storage [101]. Although emulsion stability can be improved by optimizing surfactant type and concentration, oil-to-water ratio, and adding viscosity modifiers, these formulations generally still exhibit a shorter shelf life than synthetic pesticide products [108].

A possible method for enhancing the stability and effectiveness of EO formulations is nanoemulsion technology. Because of their tiny droplet size, which minimizes gravity separation and lowers the thermodynamic driving force for coalescence, nanoemulsions—which are defined by droplet sizes generally below two hundred nanometers—exhibit improved kinetic stability when compared to traditional emulsions [109]. Furthermore, the higher surface area to volume ratio of nanodroplets may improve the bioavailability of EO components, thereby increasing their efficacy as insecticides and acaricides [101].

Several studies have shown that nanoemulsification of EOs can boost their action against ectoparasites. For instance, compared to traditional emulsions at equal doses, nanoemulsions of *Cinnamomum verum* oil had around 40% more adulticidal action against the rat flea *Xenopsylla cheopis* [109]. Similar improvements in tick control have been shown, with nanoemulsified *Rosmarinus officinalis* oil showing enhanced effectiveness and extended residual activity against *Rhipicephalus sanguineus* [110]. The mechanism underlying this enhanced activity likely involves multiple factors, such as greater penetration through the arthropod cuticle, increased stability against degradation, and modified release kinetics [101]. In addition to conventional emulsions and nanoemulsions, further advanced strategies such as microencapsulation, nanoencapsulation, and controlled-release systems offer complementary benefits to protect active components and prolong their efficacy.

Microencapsulation and nanoencapsulation methods offer further advantages by incorporating EO components into protective matrices. These matrices shield the active ingredients from environmental degradation and control their release [111]. Polymers like alginate, chitosan, and polyvinyl alcohol, lipid-based carriers including solid lipid nanoparticles and nanostructured lipid carriers, and inorganic materials like silica and clay minerals have all been studied as encapsulation materials [101,108]. Each approach has distinct advantages and limitations concerning preparation simplicity, cost, stability, release kinetics, and environmental acceptability [111].

Particularly promising for prolonging the residual action of EOs are polymer-based controlled-release formulations. Alginate-chitosan microcapsules containing thymol exhibited sustained release over fourteen days in aqueous medium. The encapsulated formulations maintained adulticidal activity against *Ctenocephalides felis* for substantially longer than non-encapsulated thymol [112]. Similarly, electrospun polymer nanofibers loaded with eugenol displayed extended-release kinetics and improved residual acaricidal action when mixed into pet bedding materials [66].

### 7.3. Novel Hybrid Approaches

A novel hybrid formulation approach combines EOs with inert desiccant dusts, such as granite rock powder. This strategy leverages the acaricidal/insecticidal properties of the oil, while the dust acts as a carrier, enhances cuticular damage, and provides a physical barrier. For example, a combination of 10% *Ocimum basilicum* (basil) essential oil with granite dust achieved 100% mortality of *Ixodes scapularis* adults within 24 h, a marked improvement over either component applied alone [113]. This synergy shows that hybrid physical-chemical formulations might boost efficacy and residual action, especially against hard-bodied ectoparasites. Traditional cedar tar (*Cedrus libani*), obtained by wood pyrolysis, has demonstrated promising insecticidal efficacy against *C. felis*, demonstrating the potential of botanical resources beyond volatile essential oils. The principal active sesquiterpenes, β-himachalene and α-atlantone, contribute to a mechanism of action unique from usual monoterpenoid-rich EOs. Pure cedar tar matched the effectiveness of 0.5% fipronil in laboratory experiments, achieving 100% flea death. While its efficacy varied geographically (LC_50_ from 8.5% to 19.5% tar concentration), likely reflecting prior exposure to synthetic pesticides in different flea populations, it highlights the potential of non-volatile plant extracts as alternative or complementary agents in ectoparasite control, potentially offering longer residual activity due to lower volatility [114].

Alongside hybrid approaches with desiccant powders or non-volatile materials, another promising strategy is to combine essential oils with synthetic insecticides/acaricides to exploit synergistic effects.

This approach offers several potential benefits: achieving effective control with lower concentrations of synthetic compounds through synergy, broadening the spectrum of activity, and potentially delaying resistance development by exerting simultaneous pressure on multiple biochemical targets [115]. Studies investigating permethrin combined with other EOs have revealed synergistic effects, permitting considerable reduction in synthetic pyrethroid concentrations [116,117]. However, regulatory considerations, toxicological studies, and customer acceptability of such hybrid goods require careful study.

### 7.4. Application-Specific Formulations

The development of spray, spot-on, collar, and shampoo formulations for companion animals must also carefully consider practical factors such as ease of application, owner compliance, aesthetic acceptance, and safety for both animals and their human caretakers [118]. Spray formulations give the advantage of full-body coverage but need large product volumes and may be poorly tolerated by some animals. Spot-on formulations, applied to a limited area of the dorsal body surface, must rely on spreading of active constituents across the animal’s surface, a process that may be insufficient for highly volatile EO components [119]. Although collar formulations can offer prolonged release, it might be difficult to distribute volatile substances evenly throughout the animal’s body [104]. Proof-of-concept for converting in vitro effectiveness into a viable product has been proven with a eugenol-based spray formulation. A spray containing 7.5% eugenol achieved 100% in vitro effectiveness against both adult *C. felis* and *R. sanguineus* larvae/nymphs within 24 h. Most notably, this formulation exhibited a sustained residual impact against fleas, maintaining 100% effectiveness for 21 days and a considerable lethal effect for up to 48 days post-application. This work marks a crucial step forward, going from basic oil-in-water emulsions to a stabilized, application-ready formulation with verified prolonged activity [57]. Application-specific formulations (sprays, spot-ons, collars) need to be carefully integrated into the broader context of integrated pest management (IPM), evaluating synergies but also potential skin and systemic interactions with synthetic products.

**Figure 1:**
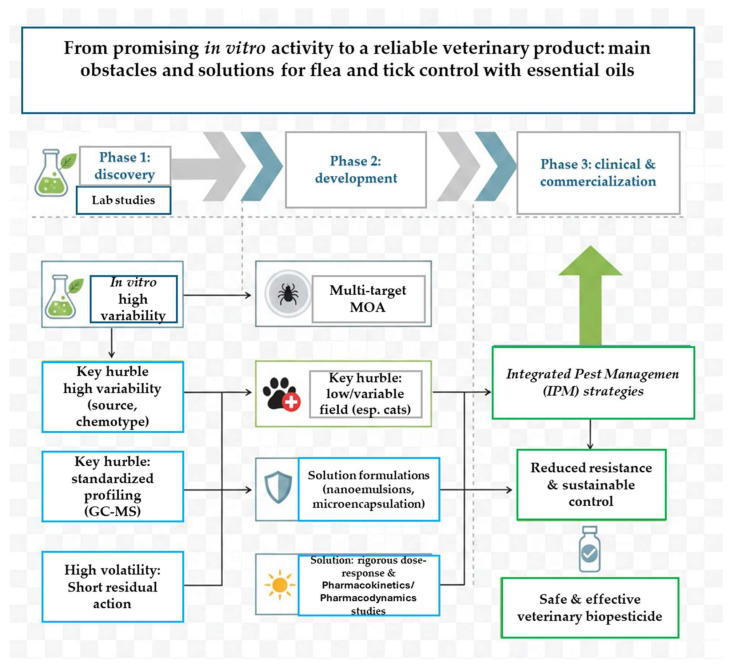
Development pathway for essential oil-based biopesticides: from laboratory discovery to integrated pest management (IPM). This flowchart illustrates the main phases of development for essential oil-based biopesticides, highlighting critical challenges (Key Hurdle) encountered and the corresponding scientific and technological solutions required (Solution) to transition from laboratory efficacy to safe and effective clinical application within Integrated Pest Management (IPM) strategies. MOA: mechanism of action; GC-MS: Gas Chromatography–Mass Spectrometry.

In the perspective of pragmatic Integrated Pest Management (IPM), EOs are increasingly considered not as total substitutes, but as potential synergists or rotation agents with established synthetic acaricides. This promising strategy, however, introduces the complex chapter of skin–drug interactions. On the one hand, there is preliminary evidence of biochemical synergy. Some monoterpenes, such as limonene, can act as inhibitors of mixed-function oxidases (P450), liver and skin enzymes involved in the detoxification of insecticides such as pyrethroids or isoxazolines [117]. This effect could theoretically enhance the action of the synthetic or overcome metabolism-based resistance mechanisms. In vitro studies on resistant *Rhipicephalus microplus* strains have shown that the combination of thymol or carvacrol with cypermethrin or deltamethrin can restore susceptibility [116]. On the other hand, the safety profile of such combinations is largely unexplored. The synergistic effect could extend to toxicity, increasing the risk of local or systemic adverse reactions. Concomitant application of two substances with potential irritant action (e.g., a phenolic OE and a fipronil-based spot-on) could compromise the skin barrier, trigger contact dermatitis, and increase unwanted systemic absorption [120]. Furthermore, for systemically acting isoxazolines (e.g., fluralaner, afoxolaner), it is not known whether the components of transdermally absorbed EOs can interfere with their binding to neuronal GABA receptors or induce metabolic enzymes that accelerate their clearance, reducing their efficacy. Therefore, before recommending “do-it-yourself” combination protocols, it is imperative to conduct preclinical studies of acute and subchronic dermal toxicity in animal models, as well as efficacy and safety trials in the field comparing the combination with individual components. Integrated management must be based on evidence of controlled and safe synergy, not on a mere overlapping of products. Figure 1 represents the principal concepts explained in this paragraph.

## 8. Safety Considerations for Target Animals

### 8.1. General Toxicity Concerns

Although EOs are often marketed as “natural” and therefore safer than synthetic alternatives, this claim requires critical examination. Depending on the particular chemicals involved, the dosage given, the mode of exposure, and individual animal characteristics, including species, age, and health state, the toxicity of EOs and their constituents to mammals, especially companion animals, varies significantly [121]. Certain EOs and their constituents can cause severe toxicity if misused, and cases of poisoning in dogs exposed to EO-containing products have been documented [107].

### 8.2. Special Risk for Cats and Documented Toxicoses

The essential oil of *Melaleuca alternifolia* (tea tree) is a well-characterized example of possible toxicity problems. Concentrated preparations or accidental ingestion have caused toxicosis in both dogs and cats, with clinical signs including depression, ataxia, tremors, and, in severe cases, coma. Notably, diluted formulations have shown acceptable safety profiles in controlled studies [122]. The mechanism entails neurotoxicity associated with monoterpenoid constituents, specifically terpinen-4-ol, and manifests more severely in cats than in dogs, a disparity primarily attributed to well-documented variations in hepatic glucuronidation capacity [123,124]. This metabolic deficit increases the risk of accumulation and toxicity following exposure to phenol-rich EOs. However, the safety profile is further complicated by the interaction of topically applied EOs with the skin’s physiology. The skin of dogs and cats is not an inert substrate; it is a dynamic, biochemical barrier. The hydrolipidic film (composed of sebum and sweat) and the slightly acidic pH (approximately 5.5–7.0 in dogs, 6.0–6.5 in cats) create a microenvironment that can rapidly alter applied EO [125]. Hydrophobic components may partition into sebum, reducing bioavailable surface concentration, while a non-neutral pH may catalyze hydrolysis or molecular rearrangements of reactive constituents. This can not only diminish acaricidal activity but also increase the potential for local irritation or sensitization [121]. Furthermore, a dense coat acts as a reservoir, absorbing a significant portion of a spray or spot-on product, thereby diverting it from direct contact with the epidermis where parasites attach and promoting its volatilization from the hair surface. This complex interaction underscores that safety and efficacy are inextricably linked to the vehicle and the animal’s cutaneous ecosystem. The need for extreme caution when evaluating EO-based treatments for feline ectoparasite control is highlighted by documented occurrences of toxicity in cats after exposure to essential oils from *Thymus*, *Origanum*, *Citrus*, *Melaleuca*, and *Pinus* species [107,126]. The risks associated with commercially available, EPA-exempt “natural” flea products that contain mixtures of essential oils (EOs) like peppermint, thyme, cinnamon, and clove oils are crucially demonstrated by a retrospective analysis of cases reported to the Animal Poison Control Center of the American Society for the Prevention of Cruelty to Animals (ASPCA) between 2006 and 2008.

Even when products were used as directed on the label, the study found that 92% of exposed animals (39 cats and 9 dogs) experienced negative impacts. Clinical symptoms included lethargy and vomiting in dogs and agitation and hypersalivation in cats. Three cases reported severe outcomes, such as euthanasia or death. This underlines the unique sensitivity of cats, underscores the fact that “natural” labeling does not equate to safety, and reveals a significant regulatory gap regarding minimum-risk pesticide products, which may not undergo thorough EPA effectiveness and safety review [107]. Conversely, some formulations have shown acceptable safety profiles in controlled studies. For example, a 3-day oral regimen of a specific EO mixture (garlic, rapeseed, rosehip oils) resulted in no adverse hematological or biochemical changes in treated dogs, supporting the principle that safety is highly formulation- and route-dependent [64].

### 8.3. Therapeutic Window and Margin of Safety

One crucial aspect of safety is the distinction between therapeutic and toxic dosages. The margin of safety, defined as the ratio between hazardous dosage and therapeutic dose, differs greatly across various EOs and their uses. For topical treatments designed for ectoparasite management, the therapeutic dosage must be adequate to kill or repel target parasites while being below quantities that induce harmful effects in the treated animal [127]. Given interindividual differences in sensitivity and the possibility of increased exposure through grooming behavior, this treatment window may be very limited for certain highly active EOs [120]. The therapeutic margin of safety is further conditioned by local skin reactions, such as irritation and sensitization, which may limit the acceptability of some essential oil products.

Additional safety issues that might restrict the acceptance of EO-based products are skin irritation and hypersensitivity. After repeated exposure, several EO compounds, especially aldehydes like citral and cinnamon aldehyde, are recognized skin sensitizers that can result in allergic contact dermatitis [128]. The irritant potential varies among different EOs and can be influenced by formulation factors, including concentration, vehicle characteristics, and the presence of penetration enhancers [127]. Although patch testing procedures have been devised to evaluate the cutaneous tolerability of EO formulations, these tests are rarely conducted before companion animal products are marketed [125].

### 8.4. Chronic Exposure Concerns

A significant knowledge gap exists regarding the safety of repeated or prolonged EO exposure, as long-term toxicity data are lacking. Most safety reviews have focused on acute toxicity following single exposures. Companion animals utilizing EO-based ectoparasite control solutions may be exposed repeatedly at monthly or more frequent intervals during their lifetimes [121]. Endocrine disruption, hepatotoxicity, and carcinogenicity are potential risks associated with long-term exposures; however, rigorous examination of these endpoints specifically for veterinary EO products is still completely lacking in the literature [129]. Concerns about chronic exposure are intertwined with quality control and standardization issues, which can amplify the variability and risks associated with prolonged use.

Issues of quality control and uniformity directly compromise product safety. The chemical composition of EOs can vary widely depending on botanical source, growth conditions, harvest time, and extraction techniques [130]. Unpredictable effectiveness and safety profiles might result from products marketed as having a certain EO, differing greatly in their constituent composition and concentrations [131]. The lack of strong legal standards for natural product standardization in many jurisdictions implies that substantial batch-to-batch variability may occur [132].

## 9. Considerations on Environmental Fate and Toxicity to Companion Animals

### 9.1. Environmental Fate and Aquatic Toxicity

A key but understudied aspect of the sustainability profile of EOs is their environmental fate and impact following application as ectoparasiticides. EOs are frequently promoted as environmentally benign alternatives to synthetic insecticides, owing to their natural origin and presumed rapid degradation, although evidence directly comparing their environmental impact to synthetic alternatives remains limited. However, a comprehensive assessment reveals a more complex picture [133]. The environmental persistence, mobility, and toxicity to non-target organisms of each component of EOs differ significantly [134]. Because topically administered ectoparasiticides can reach aquatic habitats through a variety of channels, such as bathing treated animals, surface runoff, and effluent from grooming facilities, aquatic toxicity is a major issue [135]. Some EO elements display high toxicity to aquatic invertebrates, notably crustaceans that fulfill crucial ecological roles and constitute sensitive markers of environmental pollution [136]. For instance, at concentrations in the low milligram per liter range, 1,8-cineole, a typical monoterpenoid present in eucalyptus and other oils, shows acute toxicity to *Daphnia magna* [137].

### 9.2. Terrestrial Non-Target Effects

The impact on terrestrial non-target arthropods constitutes an additional environmental concern, particularly for EO treatments sprayed outdoors for tick control or used indoors for flea management. Beneficial insects, such as pollinators, predatory arthropods, and decomposers, might be harmed by EO residues [138]. Short-term impacts on sensitive non-target species may still occur, even though the rapid volatilization of many EO constituents may limit their environmental persistence and, consequently, exposure duration for non-target organisms [139]. The selectivity of EOs for target vs. non-target arthropods requires a thorough study, but has gotten scant attention in the literature [138].

In addition to effects on non-target terrestrial arthropods, biodegradation rates and environmental persistence vary greatly as a function of the chemical structure and site conditions. Biodegradation rates of EO components in soil and water settings vary substantially depending on chemical structure, environmental circumstances, and microbial community makeup [137]. In general, soil and aquatic microorganisms facilitate oxidation and hydroxylation events that cause monoterpenoids to biodegrade rather quickly [139]. But in other environmental matrices, phenylpropanoids like eugenol show more permanence, which raises concerns about possible buildup with repeated applications [136]. The development of transition products during environmental deterioration and their potential toxicity constitute further concerns requiring inquiry [133].

### 9.3. Sustainability Assessment Frameworks

More thorough comparison with synthetic alternatives would be made possible by the creation of evaluation frameworks, particularly intended to evaluate the environmental sustainability of biopesticides, including EO-based ectoparasiticides [39]. Such frameworks should include considerations of resource usage efficiency in production, energy needs for extraction and formulation, biodegradability, acute and chronic toxicity to non-target species, and potential for bioaccumulation [140]. When compared to traditional alternatives, life cycle assessment techniques may offer insightful information about the total environmental impact of EO-based goods [141].

## 10. Regulatory and Commercialization Challenges

### 10.1. Regulatory Landscape Variability

The development and commercialization of EO-based ectoparasite control products are hindered by inconsistent regulatory frameworks across countries. In many jurisdictions, products with pesticidal claims must undergo regulatory review for efficacy and safety, irrespective of whether they contain synthetic or natural ingredients [132]. However, depending on how things are categorized and promoted, the precise standards and the level of review might differ significantly [141].

The Federal Insecticide, Fungicide, and Rodenticide Act governs pesticidal products in the US, although certain low-risk natural substances, such as some EO constituents, may be exempt from full registration requirements as “minimum risk pesticides” under certain circumstances [107]. Many EO-based flea and tick products for companion animals have entered the market due to this regulatory exemption process. However, given documented instances of adverse responses in dogs and inconsistent efficacy, questions have been raised over the sufficiency of safety and efficacy review for such exempt drugs.

The European regulatory framework, working under the Biocidal Products Regulation, demands evidence of efficacy and safety for biocidal products, including those designed for ectoparasite control, with no general exception for natural products [39]. This more strict regulatory approach has limited the commercial availability of EO-based ectoparasiticides in European countries but possibly provides stronger assurance of product quality and performance [132]. A major obstacle to entrance, especially for small businesses looking to create botanical pesticides, is the resource needs for producing data packages that satisfy regulatory standards [142].

### 10.2. Efficacy Standards and Field Performance

Efficacy criteria constitute a special problem for natural product producers. Minimum effectiveness criteria, usually between 90 and 95 percent decrease in target parasites for certain periods after treatment, are specified by regulatory standards [143]. Replicating these results in field settings with naturally infested animals has proven more challenging, even though some EO formulations have attained such efficacy levels in controlled laboratory studies [144]. The diversity in EO composition and the susceptibility to environmental variables affecting persistence may lead to inconsistent efficacy in real-world conditions [132].

### 10.3. Intellectual Property and Market Dynamics

The commercialization potential of EO-based goods is influenced by intellectual property issues. Unlike unique synthetic compounds that may be secured by composition of matter patents, individual EO ingredients and simple EO formulations may be difficult to protect, lowering the exclusivity period during which development expenditures can be recovered [141]. Proprietary formulation technologies, specialty blends, or innovative combinations may offer more defensible intellectual property positions, while the strength of such protection relative to composition patents for novel synthetic compounds remains restricted [120]. The economic feasibility of EO-based ectoparasiticides is significantly influenced by consumer perceptions and market acceptability. A positive market environment is created by customer interest in natural pesticide substitutes, but this desire must be weighed against price sensitivity and performance expectations [103]. Products that exhibit uneven performance or need more frequent application than synthetic alternatives may struggle to sustain market dominance despite their natural positioning [141]. Clear communication about acceptable expectations, constraints, and correct use is critical for developing sustainable markets for these items [58].

## 11. Current Limitations and Knowledge Gaps

### 11.1. Methodological Standardization Needs

Despite a growing body of research on EOs for ectoparasite management, our ability to fully evaluate their potential in veterinary practice is constrained by significant limitations and knowledge gaps. There is doubt regarding real-world effectiveness due to the overwhelming predominance of in vitro laboratory research compared to carefully monitored field trials [45]. The effectiveness that may be achieved under real-world use settings may be significantly overestimated by laboratory bioassays, which are usually conducted under ideal circumstances with forced contact between parasites and treated surfaces [42].

The inconsistency of methods used to assess EO activity complicates cross-study comparisons and meta-analyses. Variables that affect outcomes but are not consistently reported or controlled across research include bioassay design, parasite strain, EO source and characterization, concentration ranges investigated, exposure time, and effectiveness calculation techniques [145]. The identification of genuinely superior EOs or formulations and the development of evidence-based recommendations are hampered by this methodological variation [23]. In addition to these methodological limitations, there is a frequent lack of complete chemical characterization of the essential oils tested, which makes it difficult to correlate the observed activity to specific constituents. Many studies fail to provide a complete chemical analysis of the oils evaluated, making it impossible to relate observed activity to specific chemical constituents or to predict whether oils from the same botanical source but different suppliers or harvest conditions would show comparable efficacy [65]. Even in cases when chemical analysis is offered, it may be restricted to key elements, sometimes ignoring trace components that have a big impact on biological activity [8].

### 11.2. Pharmacological and Toxicological Data Gaps

Rational formulation development and dosage recommendations are hampered by the lack of dose–response and pharmacokinetic data. Optimization requires an understanding of the link between applied dosage, resultant surface concentrations or systemic exposures, persistence over time, and resulting effectiveness against parasites [121]. Similar to this, pharmacokinetic characterization would help safety evaluations establish the patterns of absorption, distribution, metabolism, and elimination for important EO constituents based on common exposure routes [146]. There are surprisingly few long-term, multigenerational studies looking at the possibility of EO resistance developing in ectoparasite populations. Although EO activity’s multi-target nature should, in theory, postpone the emergence of resistance, persistent selection pressure from extensive usage may result in adaptation [147]. Recommendations about proper usage patterns and the necessity of rotating with alternative control approaches would be informed by knowledge of whether and how resistance may develop [121]. Pharmacological and toxicological gaps also extend to interactions with other veterinary treatments and safety in special patient populations. There has not been much focus on how EO-based medicines interact with other veterinary treatments, including immunizations, other drugs, and therapeutic diets. Potential interactions between botanical items and drug-metabolizing enzyme systems should be thoroughly investigated [129]. Similarly, specific research is needed to determine the safety and effectiveness of EO products in certain groups, such as young animals, females who are pregnant or nursing, elderly animals, and animals with pre-existing medical disorders [148]. Table 7 summarizes the pros and cons of using essential oils as pest control agents.

## 12. Future Research Directions and Opportunities

### 12.1. Clinical Efficacy Trials

Strategic research investments addressing the aforementioned constraints are required to advance EO-based ectoparasite management toward evidence-based clinical application. There is a pressing need for well-designed, sufficiently powered field effectiveness studies that compare optimized EO formulations to existing standard-of-care treatments [142]. Such studies should employ randomized, controlled designs with sufficient power to detect clinically significant differences. They must also feature clearly defined primary and secondary efficacy endpoints, along with prolonged follow-up to assess residual activity [149]. In parallel with clinical efficacy trials in the field, the systematic study of chemotypes and the correlation between chemical profiles and biological activity is a priority to select optimized sources. The identification of better oil sources and the facilitation of quality criteria for product development may be made possible by the methodical study of EO chemotypes and the connection of chemical profiles with biological activity [37]. When metabolomics techniques are used in conjunction with bioassay-guided fractionation, it is possible to identify which particular compounds or constituent combinations are in charge of observed activity, which might result in formulations that are more effective and reliable [55]. Predictive identification of potential plant sources might be supported by chemometric modeling that links chemical composition to biological effects [150].

### 12.2. Structure–Activity Relationships

Opportunities for increasing potency, boosting selectivity for target species, or altering physicochemical features to enhance formulation characteristics may be revealed by structure–activity relationship studies looking at changes to important EO constituents [62]. Although chemically altered substances would not exactly be considered “natural”, these derivatives could nevertheless have the benefits of botanical origins, including reduced toxicity to mammals, while providing better performance [111]. Structure–activity relationship studies can guide the development of advanced controlled-release formulations, active surface platforms, and multi-compartment systems. The ability of advanced formulation methods to overcome the present constraints of EO-based products should be thoroughly evaluated. Investigation is warranted into advanced controlled-release methods, surface-active delivery platforms, and multi-compartment formulations that meet the occasionally conflicting requirements of persistence for residual killing and volatility for repellency [101]. Field performance might be enhanced by adding penetration boosters, volatilization inhibitors, or photostabilizers [151]. Exploring novel application methods, such as impregnating protective clothing or pet bedding with microencapsulated EOs, as preliminarily shown with spearmint and oregano oils against questing ticks [102], could provide passive, long-lasting protection within an IPM framework.

### 12.3. Seasonal and Strategic Application

Exploring underutilized seasons for intervention presents a novel opportunity. Research on *Abies balsamea* (balsam fir) essential oil revealed that its acaricidal activity against *I. scapularis* is significantly enhanced at low temperatures (≤4 °C), precisely when ticks are overwintering in a metabolically stressed state [152]. Because colder temperatures decrease volatilization and may enhance toxin sensitivity, applying some volatile, quickly degrading EOs in the winter may be strategically beneficial. Creating “cold-active” natural acaricides for winter environmental treatments might interrupt the tick life cycle prior to the spring increase in activity and target a vulnerable life stage with little non-target impact. Additionally, novel bioactive chemicals may be produced by systematically screening plant species from botanical groups with which important ectoparasites have had little evolutionary history (such as boreal conifers for *I. scapularis*). Seasonal and strategic approaches, molecular biomarkers, comprehensive environmental assessments, and implementation research represent further priority directions for translating the potential of essential oils into practical applications. To compare product potency and identify early indicators of decreased susceptibility, mechanism-based biomarkers for evaluating the sublethal effects of EOs on ectoparasites may be developed [117]. Molecular techniques analyzing the transcriptome, proteomic, or metabolomic responses of exposed parasites may identify indicators of successful treatment and targets for tracking the emergence of resistance [136].

To support claims of environmental superiority over synthetic alternatives, thorough environmental fate and consequences investigations are required. Standardized ecotoxicological testing procedures with pertinent non-target species at various trophic levels, evaluation of transformation product production and toxicity, and assessment of biodegradation rates under ecologically relevant circumstances should all be used in such investigations [140]. A comprehensive assessment of the environmental effects of manufacturing, usage, and final environmental destiny might be obtained through comparative life cycle evaluations [153].

Lastly, implementation research that looks at factors impacting owner adherence to treatment recommendations, cost-efficiency, and real-world effectiveness would offer important insights for converting efficacy into impact. The advantages of EO-based products for animal welfare and public health might be maximized by identifying obstacles to their proper usage and creating solutions.

## 13. Conclusions

Essential oils and their bioactive constituents represent a scientifically grounded, though not yet clinically mature, class of botanical alternatives for companion animal ectoparasite control. The evidence reviewed here confirms that phenolic monoterpenoids (carvacrol, thymol) and phenylpropanoids (cinnamaldehyde, eugenol) exert potent in vitro acaricidal and insecticidal activity via multi-target mechanisms, and that advanced formulation technologies—nanoemulsions, microencapsulation, and polymer matrices—can substantially extend residual efficacy. However, a critical and persistent gap exists between laboratory promise and field performance, driven by EO volatility, compositional variability, and the paucity of rigorous in vivo data. Safety concerns, particularly the well-documented risk of phenol-related toxicity in cats, further constrain direct clinical application without species-specific precautions. For clinicians and veterinary practitioners, the actionable takeaway is clear: EO-based products should currently be positioned as adjuncts within structured IPM programs—not as standalone replacements for registered ectoparasiticides—with product selection guided by species-specific safety data, chemotype-validated formulations, and transparent label claims. For researchers, the priority agenda includes: randomized controlled field trials comparing optimized EO formulations against standard-of-care synthetics; pharmacokinetic characterization of key constituents in dogs and cats; long-term resistance monitoring under field-use conditions; and development of harmonized bioassay standards to enable meaningful cross-study comparisons. Progress on these fronts will determine whether the theoretical advantages of EOs—multi-target mechanisms, lower environmental persistence, and broad consumer acceptability—can be reliably translated into tools that measurably improve animal welfare and reduce the burden of vector-borne disease within the One Health framework.

## Figures and Tables

**Table 1:** In vitro efficacy of essential oils and components against fleas (*Ctenocephalides felis felis*).

Target Species (Parasite)	Assay Method	Essential Oil/Component	Main Component (If Specified)	LC50/LC90 or % Efficacy	Tested Concentrations	Reference
*Ctenocephalides felis felis* (adults, eggs, larvae)	Filter paper impregnation test	*Alpinia zerumbet*, *Cinnamomum* spp., *Laurus nobilis*, *Mentha spicata*, *Ocimum gratissimum*, *Cymbopogon nardus*	*O. gratissimum*: eugenol (74.5%); *Cinnamomum* spp.: (E)-cinnamaldehyde (91.7%); *M. spicata*: carvone (83.3%)	LC50 (adults, 24 h): *O. gratissimum* 5.85 μg/cm^2^; *Cinnamomum* spp. 67.87 μg/cm^2^; LC50 (eggs): *O. gratissimum* 1.79 μg/cm^2^; LC50 (larvae): *O. gratissimum* 1.21 μg/cm^2^.	1.56–800 μg/cm^2^	[23]
*Ctenocephalides felis felis* (adults, eggs)	Filter paper impregnation test (adults, eggs), Cytotoxicity test on *S. cerevisiae*	*Schinus molle*–Extracts (hexane, ethyl acetate, methanol) and essential oils (leaves, fruits)	Hexane extract: lupenone (50.3%); Leaf oil: spathulenol, cubenol; Fruit oil: 4-terpineol (18.5%), myrtenal (20.9%)	LC50 (adults, leaf oil): 12.02 μg/cm^2^; LC50 (adults, fruit oil): 353.95 μg/cm^2^. Efficacy (eggs): not active. Cytotoxicity: low at 200 μg/mL.	Adults: 1.56–800 μg/cm^2^; Eggs: up to 800 μg/cm^2^; Cytotoxicity: 78–1250 μg/mL	[65]
*Ctenocephalides felis felis* (eggs, larvae, pupae, adults)	Filter paper impregnation test, Toxicity test on *S. cerevisiae*	*Baccharis trimera*, *Mimosa verrucosa*	*B. trimera*: carquejyl acetate (33.0%), carquejol (7.1%); *M. verrucosa*: β-pinene (14.2%), (E)-caryophyllene (13.8%), α-pinene (10.6%)	LC90 (adults): *B. trimera* 678.1 μg/cm^2^; *M. verrucosa* 678.1 μg/cm^2^. LC90 (larvae): *B. trimera* 44.9 μg/cm^2^; *M. verrucosa* 731.3 μg/cm^2^. Residual effect: 3 days.	50–2000 μg/cm^2^	[66]

**Note:** Concentration units are presented unchanged from the source publications to preserve fidelity. Differences reflect assay methodology: immersion/larval packet tests commonly report % (*w*/*v*), ppm, or mg/mL; filter-paper/contact assays report μg/cm^2^. Readers should consult individual references for exact definitions.

**Table 4:** In vitro studies on the development and evaluation of formulations based on essential oils.

Target Species (Parasite)	Assay Method	Essential Oil/Component	Main Component (If Specified)	LC50/LC90 or % Efficacy	Tested Concentrations	Reference
*Rhipicephalus sanguineus* (adults)	Formulation development (spray and spot-on), In vitro test (knockdown and residual effect)	Eugenol and Carvacrol (10% in formulations)	Eugenol, Carvacrol	Knockdown (ticks): Spot-on (combination) 100% in 6 h; Spray (combination) 100% in 12 h. Residual efficacy (ticks): only 2–3 days.	10% (*v*/*v*) bioactives	[66]
*Rhipicephalus sanguineus*	Adult Immersion Test (AIT), In vitro cytotoxicity test	*Commiphora myrrha*, *Pogostemon cablin*, *Cupressus sempervirens*—oils and nanoemulsions	Not specified	LC50 (oils, 5 days): Myrrh 9.01%; Patchouli 12.40%; Cypress 15.21%. LC50 (nanoemulsions, 7 days): Myrrh 4.17%; Cypress 5.04%; Patchouli 8.57%.	Oils: 7.5–60%; Nanoemulsions: 2.5–20%	[81]

**Note:** Concentration units are presented unchanged from the source publications to preserve fidelity. Differences reflect assay methodology: immersion/larval packet tests commonly report % (*w*/*v*), ppm, or mg/mL; filter-paper/contact assays report μg/cm^2^. Readers should consult individual references for exact definitions.

**Table 7:** Critical analysis: opportunities versus challenges for the practical application of essential oils in veterinary ectoparasite control.

Opportunity/Potential Advantage	Associated Challenge/Current Reality	Key Research & Development Need
1. Multi-Target Mechanisms of Action (Neurotoxicity, cuticle disruption, respiratory inhibition).	Slower resistance development has been theorized but not yet proven in field conditions. Detoxification enzyme induction in ticks is documented.	Long-term resistance monitoring studies under field-selection pressure.
2. Lower Environmental Persistence & Mammalian Toxicity (compared to some synthetics).	“Natural” does not equal safe. Documented adverse events; high species-specific risk (cats).	Standardized safety protocols (acute & chronic) for target species; clear regulatory pathways.
3. Activity Across Parasite Life Cycle (e.g., ovicidal, larvicidal effects).	Stage-specific susceptibility: Pupae and eggs are often more resistant, requiring prolonged residual activity.	Formulations that combine immediate kill (adulticide) with long-term growth disruption (IGR-like effects).
4. Source of Novel Bioactive Compounds for new product development.	Extreme variability in oil composition (chemotype, harvest, processing) leads to inconsistent efficacy.	Standardization & chemotype selection based on bioactivity; development of isolated, stabilized actives (e.g., eugenol).
5. Role in Resistance Management & IPM as a rotational agent or synergist.	Efficacy “Gap”: Potent in vitro activity often fails to translate to reliable field performance on animals.	Advanced formulation science (nanoemulsions, encapsulation) to enhance stability, residual activity, and skin distribution.
6. Consumer Demand for “Green” Alternatives.	Lack of robust, independent field efficacy trials comparing optimized EO products to standard-of-care synthetics.	Rigorous, randomized controlled field trials with clear endpoints (e.g., ≥90% efficacy for 4 weeks).

## Data Availability

No new data were created in this study.
